# Chaetocin exhibits anticancer effects in esophageal squamous cell carcinoma via activation of Hippo pathway

**DOI:** 10.18632/aging.204801

**Published:** 2023-06-14

**Authors:** Lin Li, Hangyu Jiang, Yuqi Li, Xiaochong Xiang, Yueming Chu, Jie Tang, Kang Liu, Danqun Huo, Xiaofen Zhang

**Affiliations:** 1 Department of Pharmacy, The Second Clinical Medical College of North Sichuan Medical College, Nanchong, China; 2 Key Laboratory for Biorheological Science and Technology of Ministry of Education, Bioengineering College of Chongqing University, Chongqing, China; 3 School of Pharmacy, North Sichuan Medical College, Nanchong, China; 4 Department of Pharmacy, Nanchong Traditional Chinese Medicine Hospital, Nanchong, China; 5 Institute of Tissue Engineering and Stem Cells, The Second Clinical Medical College of North Sichuan Medical College, Nanchong, China; 6 Nanchong Key Laboratory of Individualized Drug Therapy, Nanchong, China

**Keywords:** esophageal squamous cell carcinoma, chaetocin, ROS, Hippo pathway, apoptosis

## Abstract

Dysfunction of the Hippo pathway is common in esophageal squamous carcinoma (ESCC). Chaetocin, a small molecular compound isolated from the marine fungus, exhibits potent anticancer effects. However, the anticancer effects of chaetocin on ESCC and its potential relationship to Hippo pathway remain unclear. Here, we demonstrated that chaetocin dramatically inhibited the proliferation in ESCC cells by causing cycle arrest in the M phase and activating the caspase-dependent apoptosis signaling pathway *in vitro*, and we also found that chaetocin induced the accumulation cellular reactive oxygen species (ROS). The RNA-seq analysis indicated that the Hippo pathway is one of the most enriched pathways after chaetocin treatment. We further revealed that chaetocin triggered the activation of Hippo pathway in ESCC cells, which is characterized by elevated phosphorylation levels of almost all core proteins in Hippo pathway, such as MST1 (Thr183), MST2 (Thr180), MOB1 (Thr35), LAST1 (Thr1079 and Ser909) and YAP (Ser127), ultimately leading to decreased nuclear translocation of YAP. Moreover, the MST1/2 inhibitor XMU-MP-1 not only partially rescued the inhibitory effect chaetocin-induced proliferation, but also rescued the chaetocin-induced apoptosis in ESCC cells. Furthermore, *in vivo* results confirmed the antitumor effect of chaetocin and its relationship with Hippo pathway. Taken together, our study demonstrates that chaetocin exhibits anticancer effects in ESCC via activation of Hippo pathway. These results provide an important basis for further research of chaetocin as a potential candidate for ESCC treatment.

## INTRODUCTION

Esophageal cancer (EC) is reported to be the seventh most common cancer worldwide and the sixth cancer in terms of mortality overall in 2020 [1]. Esophageal squamous cell carcinoma (ESCC) is the predominant histological type of EC, especially in China and other East Asian countries [2]. Although the overall survival of EC has improved in recent decades due to medicine development, the 5-year overall survival rate of ESCC remains at only 15–25% [3, 4]. The main causes of treatment failure are recurrence and metastasis concurrent with treatment resistance [4, 5]. It is thus critical to identify novel agents with better efficacy for ESCC treatment.

The Hippo pathway, originally discovered in Drosophila, was an extremely evolutionarily conserved pathway [6]. The core components of Hippo pathway of mammal consist of MTS1/2, LATS1/2, SAV1, MOB1, YAP, TAZ, and TEADs [7]. Dysfunction of the Hippo pathway is common and controls multiple biological processes in cancer, including tumorigenesis, aggressiveness, invasion, migration, metastasis [7, 8]. Compared with normal mucosa, frequent inactivation mutations of Hippo pathway have been confirmed and validated in ESCC [4, 9, 10]. Increasing evidence suggests that the upstream core kinases of Hippo pathway act as tumor suppressors whereas downstream YAP/TAZ as oncogenes, such as ESCC [11–13]. Several studies have revealed that YAP/TAZ were overexpressed and promoted cell proliferation, invasion, migration, metastasis and drug resistance in ESCC [11, 12, 14]. Therefore, the Hippo pathway plays a pivotal role in ESCC, and the pathway has become an attractive target for therapeutic development. Recent research has pointed out how the Hippo pathway and oxidative stress are related and illustrated how ROS might stimulate the Hippo pathway [15]. Therefore, some small molecule targeting ROS/Hippo is being investigated. It was reported that alantolactone suppresses YAP1/TAZ by stimulating the generation of reactive oxygen species, thus suppressing the growth of tumors [16].

Chaetocin is a small molecular compound isolated from the marine fungus genus Chaetomium. Previous studies have revealed that chaetocin has multiple pharmacological activities, such as antiviral, antiparasitic, anti-gout and cardiovascular protective effects [17–20]. We have reviewed recent studies showing that chaetocin exhibits potent inhibitory effects against a variety type of cancers [21–23]. However, the effect of chaetocin against ESCC cells and the underlying mechanism need further elucidation.

Here, we attempt to explore the inhibitory effect of chaetocin on the biological function of ESCC cells. Moreover, whether its molecular mechanism is associated to the activation of Hippo pathway was further explored. These results provide an important basis for further research of chaetocin as a potential therapeutic candidate for ESCC treatment.

## MATERIALS AND METHODS

### Cell lines and cell culture

Human ESCC cell lines (KYSE150, Eca109, KYSE30, KYSE70, KYSE410, KYSE510, TE-1, TE-11) and normal esophageal epithelial cell (Het-1A) were provided by Institute of Tissue Engineering and Stem Cells (Nanchong, China). Seven ESCC cell lines (Eca109, KYSE30, KYSE150, KYSE410, KYSE510, TE-1, TE-11) were cultured in RPMI-1640 supplemented with 10% fetal bovine serum (FBS). Het-1A and KYSE70 were cultured in DMEM medium supplemented with 10% FBS. All cells were then incubated in a humidified atmosphere of 5% CO_2_ at 37°C.

### Reagents and antibodies

Chaetocin (Cat. HY-N2019-10 mg), N-acetyl-L-cysteine (NAC) (Cat. HY-B0215-500 mg), XMU-MP-1 (Cat. HY-100526-5 mg), z-VAD-fmk (Cat. HY-16658B-5 mg) and Necrostatin-1 (Cat. HY-15760-10 mg) were purchased from MedChemExpress (Shanghai, China). The anti-p-Histone h3 antibody (Cat. ET1601-30), anti-PARP antibody (Cat. ET1608-56), anti-Cleaved PARP antibody (Cat. ET1608-10), anti-Caspase-3 antibody (Cat. ET1608-64), anti-Cleaved-Caspase-3 antibody (Cat. ET1608-64), anti-Bcl-2 antibody (Cat. ET1702-53) and anti-Bax antibody (Cat. ET1603-34) were purchased from HuaBio (Hangzhou, China). The anti-p-CDK1 (Thr161) antibody (Cat. BS-3481R) was purchased from BIOSS (Beijing, China). The anti-MST1 antibody (Cat. 3682), anti-MST2 antibody (Cat. 3952), anti-p-MST1 (Thr183)/MST2 (Thr180) antibody (Cat. bs-3294R), anti-LATS1 antibody (Cat. 3477), anti-p-LATS1 (Ser909) antibody (Cat. bs-3246R), anti-p-LATS1 (Thr1079) antibody (Cat. bs-3245R), anti-MOB1 antibody (Cat. 13730), anti-p-MOB1 (Thr35) antibody (Cat. 8699), anti-SAV1 antibody (Cat. 13301), anti-YAP antibody (Cat. 14074), anti-p-YAP (Ser127) antibody (Cat. 13008), anti-p-YAP (Ser397) antibody (Cat. 13619) and GAPDH (Cat. 5174) were purchased from Cell Signaling Technology (Danvers, MA, USA).

### Cell viability assay

TE-1, KYSE150, Eca109, KYSE410, KYSE510, KYSE70, TE-11, KYSE30 and Het-1A (all at 3 × 10^4^ cells/well) were seeded in 96-well plates, and treated with various concentration of chaetocin for 24 h. CCK-8 assay was then performed according to our previous study [24].

### Colony formation assay

TE-1 and KYSE150 cells were seeded in a 6-well plate (500 cells/well) and treated with various concentration of chaetocin. Cells were cultured in a 5% CO_2_ atmosphere at 37°C for 7 d, and the culture medium was changed every 3 d. After chaetocin treatment, cells were washed with PBS, fixed with 4% paraformaldehyde, and stained with 0.5% crystal violet. Then the colonies were imaged and counted.

### EdU staining assay

The EdU-594 Detection Kit was purchased from the Beyotime Biotechnology (Shanghai, China). TE-1 and KYSE150 cells were seeded in 24-well plates at a density of 1 × 10^4^ cells/well, and treated with different concentrations of chaetocin (0, 0.2, 0.4, 0.8 μM) for 24 h. Then, 20 μM EdU was added and incubated at 37°C for 2 h to label the cells. Next, 4% paraformaldehyde was added to fix the cells, and 200 μL 0.5% Triton X-100 was added to each well for 10 min to increase cell permeability. After the above operation, the cells were washed with PBS for 3 times, each well was added with 500 μL click reaction solution, and incubated for 30 min in the dark. The cells were washed three times again, and 500 μL Hoechst 33342 was added to each well to stain the nuclei at room temperature for 10 min. Finally, the samples were analyzed and imaged.

### Cell cycle analysis

TE-1 and KYSE150 cells (at 2 × 10^5^ cells/well) were seeded in 6-well plates, and treated with chaetocin at different concentrations (0, 0.2, 0.4, and 0.8 μM) for 24 h. Cell cycle analysis was then performed according to our previous study [24].

### Cell apoptosis assay

The Annexin V-FITC/PI Apoptosis Kit was purchased from Vazyme Biotech (Nanjing, China). TE-1 and KYSE150 cells were seeded in 6-well plates at a density of 2 × 10^6^ cells/well, and treated with chaetocin at different concentrations (0, 0.2, 0.4, and 0.8 μM) for 24 h in the presence or absence of NAC (5 mM) or z-VAD-fmk (10 μM) before collection. The cells were analyzed by flow cytometry as previously described [25].

### ROS measurement

The ROS Assay Kit was purchased from Keygen Biotech (Jiangshu, China). TE-1 and KYSE150 cells were incubated into 6-well plates at 1 × 10^5^ cells/well, treated with chaetocin at different concentrations (0, 0.2, 0.4 and 0.8 μM) for 24 h. Then, the upper suspension cells and adherent cells were collected, added with 10 μM 2′,7′-dichlorofluorescin diacetate (CM-H2DCFDA) into each cell, and incubated at 37°C for 30 min in the dark. The cells were then resuspended in 500 μL of PBS. Finally, fluorescence measurements were performed using flow cytometry (BD Biosciences, Franklin Lakes, NJ, USA).

### Western blot analysis

TE-1 and KYSE150 cells were incubated into 10 cm plates, treated with chaetocin at different concentrations (0, 0.2, 0.4, 0.8 μM) for 24 h. Then, the cells were collected, a mixture of RIPA buffer and protease inhibitor was added. The total protein was separated via sodium dodecyl sulfate polyacrylamide gel electrophoresis and transferred to a PVDF membrane. After soaking and shaking with 5% non-fat milk for 1 h, the PVDF membranes were incubated with primary antibodies overnight at 4°C. On the second day, the primary antibody was recovered and the secondary antibody was coupled by HRP. Incubation was done at room temperature for 1 hour for ECL detection and the film was developed.

### Immunohistochemistry

The tumor was removed 14 days after administration, fixed with 10% formaldehyde and embedded in paraffin. Then, the paraffin-embedded tissue was cut into 3-μm sections. The sections were roasted for 20–30 minutes to evaporate the water and melt the paraffin, the antigen retrieval with 0.01 M sodium citrate buffer (pH 6.0). After adding 3% hydrogen peroxide for 15 min, sections were added with 0.1% Triton X-100 and incubated for 10 min. After 3% BSA blocking, the primary antibody Ki67, cleaved caspase-3, YAP and p-YAP were incubated for 2 h, followed by HRP secondary antibody at room temperature for 1 h. At last, the sections were visualized with 3,3′-diaminobenzidine tetrahydrochloride (DAB) imaging section and counterstained with hematoxylin.

### Xenograft mouse model

A total of 16 male BALB/c nude mice (age, 4–6 w; weight, 18–22 g) were purchased from the Academy of Military Medical Science (Beijing, China). The mice were maintained in a pathogen-free environment at 22–26°C with sufficient food and water. Following 1 w of adaptation, KYSE150 cells (5 × 10^6^ cells in 150 μL PBS) were implanted into the right flanks of the nude mice. Once the tumor sizes reached approximately 100 mm^3^, tumor-bearing mice with similar tumor volumes were randomly grouped (*n* = 8 per group). Groups were assigned to intraperitoneally receive the vehicle (2% DMSO) or chaetocin (0.5 mg/kg/d) groups for 14 d prior to sacrifice. Tumor volumes were measured using a caliper and calculated using the formula: Volume (mm^3^) = width^2^ × length × 0.5. Tissue samples were collected and processed for further analysis. The tumors were then collected and analyzed by immunohistochemistry and Western blot. The heart, liver, spleen, lung, and kidney were dissected and fixed with 4% polyformaldehyde and stained with hematoxylin-and-eosin at room temperature for 1 min for further histological confirmation.

### RNA-seq assay

TE-1 cells were inoculated into a 6-cm culture dish at a density of 2.5 × 10^5^ cells/well during the exponential growth phase, and treated with chaetocin (0, 0.4, 0.8 μM) for 24 h (*n* = 2). The cells were digested by Trizol according to the standard protocol, then collected and transferred to −80°C for storage. The following steps including RNA isolation, cDNA library preparation and RNA-seq were completed by Beijing Genomics Institute (Beijing, China).

### Statistical analysis

Data were analyzed by SPSS 20.0 (Chicago, IL, USA) and graphs were generated by GraphPad Prism 6.0 (San Diego, CA, USA). All data were derived from at least three replicated independent experiments. One way analysis of variance (ANOVA) or Student’s *t* test was performed for statistical analyses. The following terminology was considered statistically significant: ^*^P < 0.05, ^**^P < 0.01, ^***^P < 0.001, ^****^P < 0.0001.

## RESULTS

### Chaetocin suppresses the growth of ESCC cells

**Figure 1:**
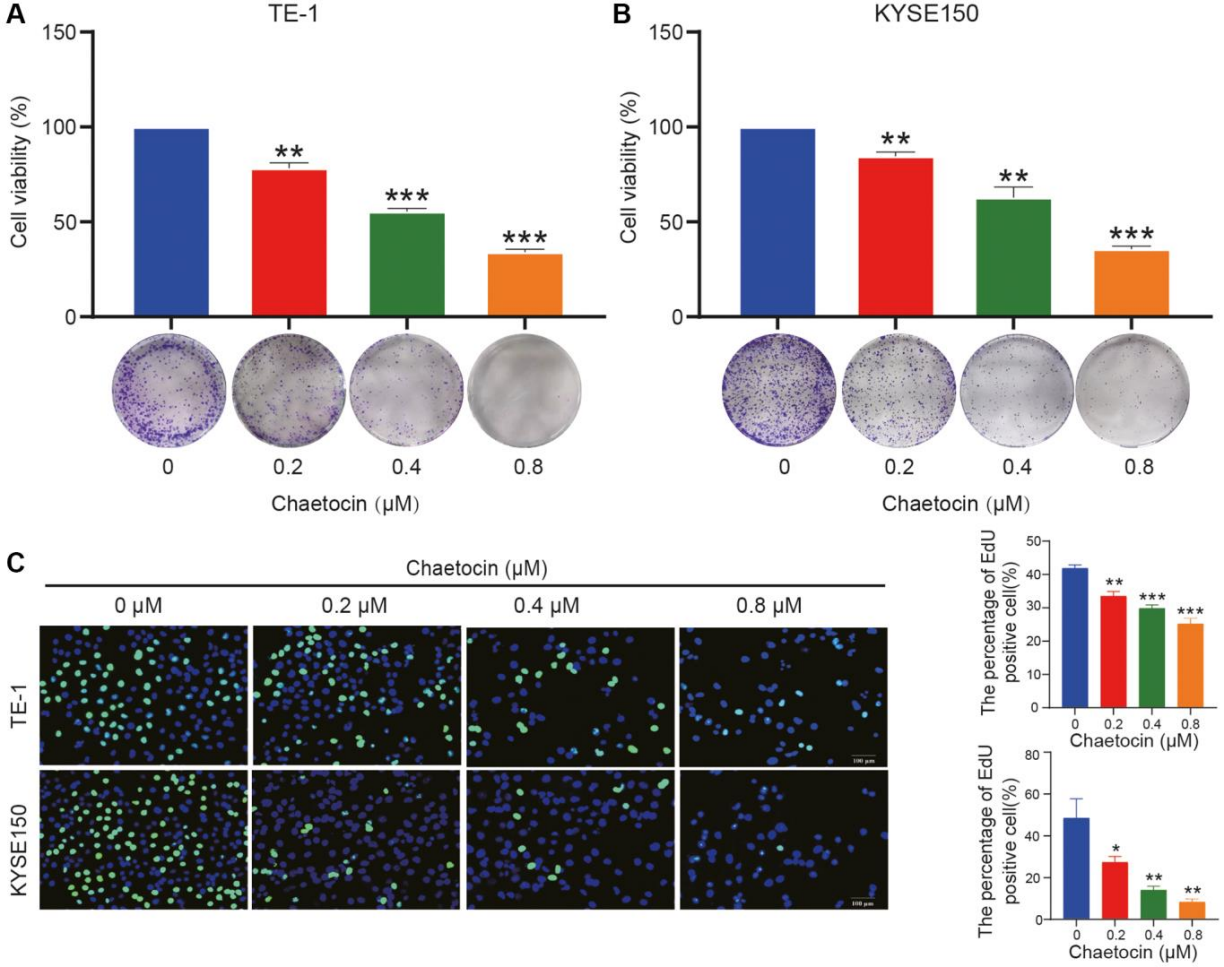
**Chaetocin suppresses the growth of ESCC cells.** (**A**, **B**) TE-1 and KYSE150 cells were treated with the indicated concentrations of chaetocin (0, 0.2, 0.4, and 0.8 μM) for 24 h. The cell viability was examined by CCK-8. The colony formation was evaluated after 14 days. (**C**) EdU assay was used to measure cell proliferation. The results are presented as the mean ± SD and are representative of at least three independent experiments. ^*^P < 0.05, ^**^P < 0.01, ^***^P < 0.001 compared with the control group.

Chaetocin is a fungal metabolite with a thiodioxopiperazine structure (Supplementary Figure 1A). We first investigated the cytotoxic effects of chaetocin on various ESCC cell lines (TE-1, TE-11, KYSE30, KYSE70, KYSE150, KYSE410, KYSE510, ECa109) and a normal esophageal epithelial cell line (Het-1A). Cells were treated with different concentrations of chaetocin for 24 h and cell viability was assayed using a CCK-8 assay. Chaetocin treatment significantly reduced ESCC cell viability, and the growth-suppressing effect of chaetocin was more pronounced against ESCC cells than normal Het-1 A cell (Supplementary Figure 1B and Supplementary Table 1). Surprisingly, IC_50_ values of chaetocin to ESCC cells were lower than those of cisplatin (Supplementary Figure 1C and Supplementary Table 2), which is one of the most effective chemotherapeutic agents currently used to treat ESCC. TE-1 and KYSE150 cells were selected for subsequent studies, and chaetocin reduced ESCC cell viability in a dose-dependent manner (Figure 1A, 1B).

Next, we evaluated the effect of chaetocin on the formation of ESCC cell clones. TE-1 and KYSE150 cells were pretreated with different concentrations of chaetocin for 24 h, chaetocin reduced colony formation in a dose-dependent manner (Figure 1A, 1B). In addition, the anti-proliferative activity of chaetocin was evaluated using an EdU assay. Chaetocin significantly suppressed the proliferation of both TE-1 and KYSE150 cells (Figure 1C). Taken together, these results demonstrate that chaetocin inhibits the proliferation of ESCC cells *in vitro*.

### Chaetocin triggers mitotic arrest in ESCC cells

**Figure 2:**
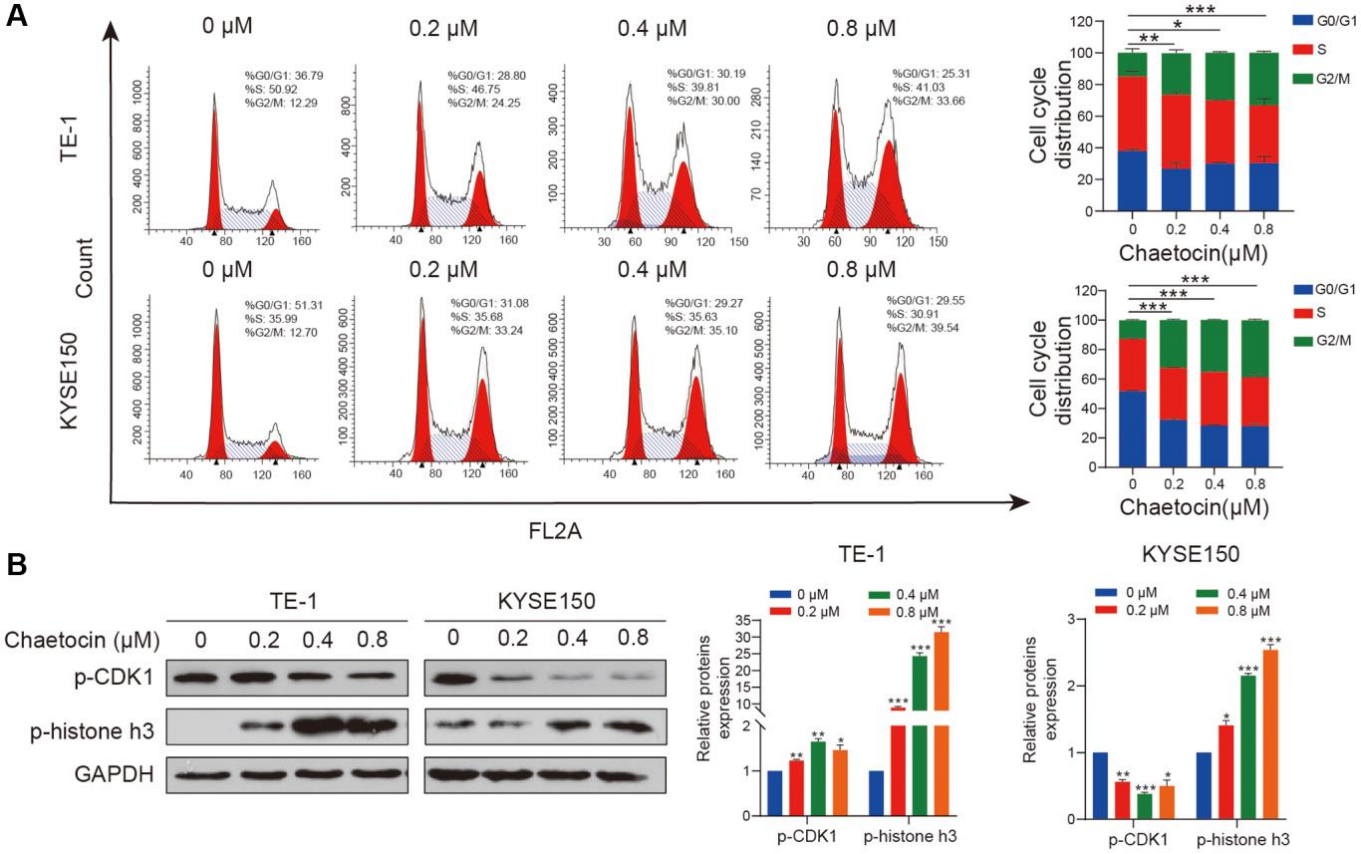
**Chaetocin triggers mitotic arrest in ESCC cells.** (**A**) TE-1 and KYSE150 cells were treated with the indicated concentrations of chaetocin (0, 0.2, 0.4 and 0.8 μM) for 24 h. The cell cycle distribution was analyzed using flow cytometry. The values indicate the mean ± SD of three independent experiments. ^*^P < 0.05, ^**^P < 0.01, ^***^P < 0.001 compared with the control (one-way analysis of variance). (**B**) Protein expression of p-CDK1 and p-histone h3 was detected using western blot analysis. GAPDH was used as the loading control. Blots presented here are representative of three independent experiments. ^*^P < 0.05, ^**^P < 0.01, ^***^P < 0.001 compared with the control group.

To determine whether chaetocin induces cell cycle arrest in ESCC cells, we performed cell cycle distribution analyses using flow cytometry. As shown in Figure 2A, after pretreatment with different concentrations of chaetocin for 24 h, the population of TE-1 and KYSE150 cells in the G2/M phase increased in a concentration-dependent manner compared with untreated cells, indicating that chaetocin inhibits ESCC cell proliferation by prompting G2/M phase arrest. To determine whether chaetocin induces ESCC cell cycle arrest in G2 or M phase, we further analyzed the expression of p-CDK1 and p-histone h3 using Western blotting. TE-1 and KYSE150 cells pretreated with different concentrations of chaetocin for 24 h exhibited a concentration-dependent decrease in the expression of p-CDK1, whereas the expression of p-histone h3 was increased with increasing chaetocin concentration (Figure 2B), indicating that chaetocin causes mitotic arrest in ESCC cells.

### Chaetocin induces ESCC cells apoptosis in a Caspase-dependent manner

**Figure 3:**
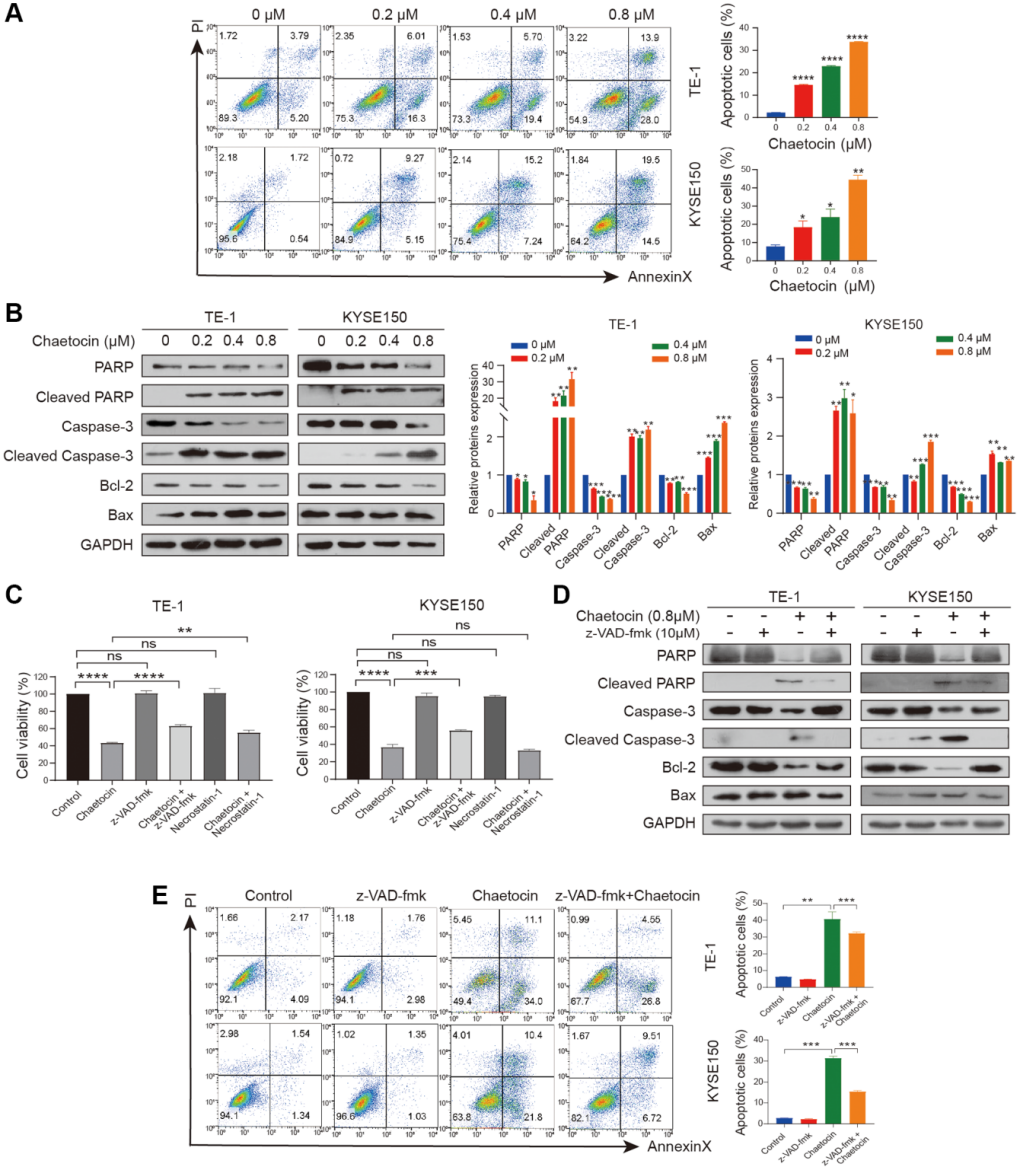
**Chaetocin induces ESCC cells apoptosis in a caspase-dependent manner.** (**A**) TE-1 and KYSE150 cells were treated with the indicated concentrations of chaetocin for 24 h, and apoptotic rates were detected using Annexin V/PI staining and flow cytometry. Results are shown as mean ± SD of three independent experiments. ^*^P < 0.05, ^**^P < 0.01, ^***^P < 0.001, ^****^P < 0.0001 vs. control group. (**B**) Western blot analysis of PARP, Cleaved PARP, caspase-3, cleaved-caspase-3, Bax and Bcl-2 following treatment with 0–0.8 μM chaetocin for 24 h. GAPDH was utilized as an internal standard. Blots presented here are representative of three independent experiments. ^*^p < 0.05, ^**^p < 0.01, ^***^p < 0.001 compared with the control group. (**C**) TE-1 and KYSE150 cells were pretreated with Z-VAD-FMK (10 μM, 2 h) or necrostatin-1 (20 μM, 2 h) before chaetocin treatment (0.8 μM, 24 h), and the cell viability was analyzed by CCK8 assay. (**D**) Expression levels of PARP, Cleaved PARP, caspase-3, cleaved-caspase-3, Bax and Bcl-2 were detected by western blot. GAPDH was used as the loading control. (**E**) Apoptosis was analyzed by flow cytometry. Results in (**C**) and (**E**) are shown as mean ± SD of three independent experiments. ^**^P < 0.01, ^***^P < 0.001, ^****^P < 0.0001, ns nonsignificant compared with the control group.

We next examined whether chaetocin could induce apoptosis in ESCC cells. TE-1 and KYSE150 cells were treated with different concentrations of chaetocin for 24 h, after which apoptosis was assessed using an Annexin V-FITC/PI apoptosis assay. Compared with the control group, chaetocin treatment promoted the population of early and late apoptosis of ESCC cells in a dose-dependent manner (Figure 3A). To further confirm that chaetocin induced apoptosis of ESCC cells, the expression of apoptosis-related proteins was analyzed by Western blotting. As shown in Figure 3B, PARP and caspase-3 expression was activated by chaetocin treatment, and levels of the cleaved forms of these proteins increased in a concentration-dependent manner. Western blot analysis demonstrated that levels of the anti-apoptotic proteins BCL-2 decreased and the pre-apoptotic proteins BAX increased following chaetocin treatment. These results suggest that chaetocin induces apoptosis in ESCC cells.

To determine whether the chaetocin-induced apoptosis of ESCC cells is caspase-dependent, cells were treated with pan-caspase inhibitor z-VAD-fmk and chaetocin to induce apoptosis. However, treatment with the pan-caspase inhibitor z-VAD-fmk only partially suppressed chaetocin-induced apoptosis. As showed in Figure 3C, chaetocin did not induce necroptosis, as treatment with the necroptosis inhibitor necrostatin-1 had no effect on chaetocin-induced death of TE-1 and KYSE150 cells. In cells treated with the z-VAD-fmk and then chaetocin to induce apoptosis, z-VAD-fmk significantly blocked chaetocin-induced caspase pathway activation (Figure 3D). The apoptotic ESCC cell population following treatment with chaetocin was partially restored by z-VAD-fmk treatment (Figure 3E), indicating that chaetocin induces apoptosis in ESCC cells via the caspase pathway.

### Chaetocin induced the accumulation of ROS in ESCC cell

**Figure 4:**
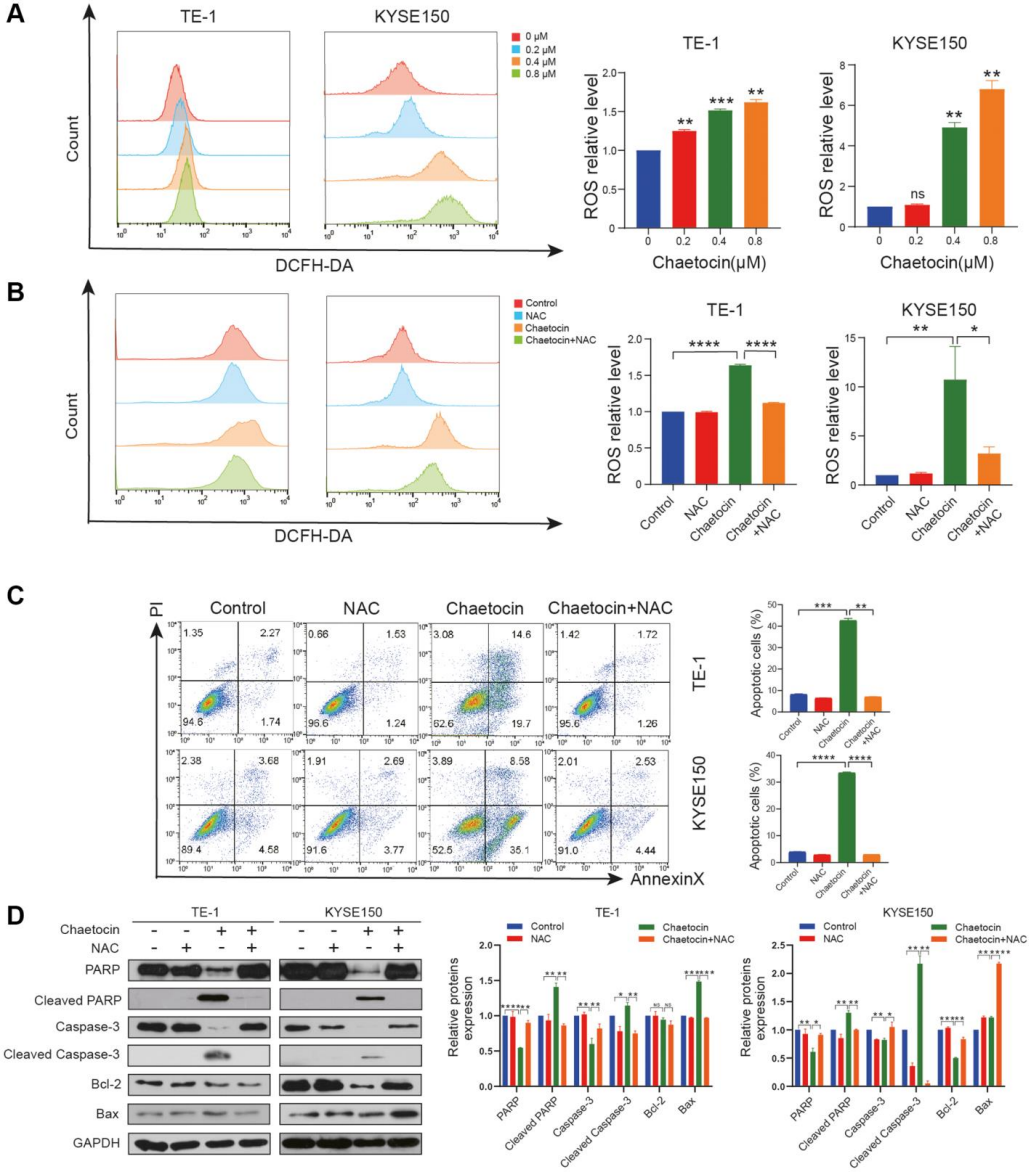
**Chaetocin induced the accumulation of ROS in ESCC cell.** (**A**, **B**) ROS levels were analyzed by flow cytometry in TE-1 and KYSE150 cells. Results were shown as mean ± SD of three independent experiments. ^*^P < 0.05, ^**^P < 0.01, ^***^P < 0.001, ^****^P < 0.0001, ns nonsignificant compared with the control group. TE-1 and KYSE150 cells were pretreated with 5 mM NAC for 1 h and then cotreated with chaetocin (0.8 μM). (**C**) Apoptosis was measured by flow cytometry. Results were shown as mean ± SD of three independent experiments. ^**^P < 0.01, ^***^P < 0.001, ^****^P < 0.000. (**D**) Expression levels of PARP, Cleaved PARP, caspase-3, cleaved-caspase-3, Bax and Bcl-2 were detected by western blot. GAPDH was used as the loading control.

It has been previously reported that chaetocin induces apoptosis in gastric cancer and glioma cells through the accumulation of ROS [26, 27]. In the present study, analyses using a fluorescent DCFH/DA probe revealed that ROS levels in ESCC cells increased significantly upon chaetocin treatment (Figure 4A). Then, the cells were co-treated with NAC, a ROS scavenger, to inhibit the production of ROS. Subsequent flow cytometry analyses indicated that the accumulation of ROS induced by chaetocin in ESCC cells was almost completely reversed by NAC (Figure 4B). Flow cytometry also showed that NAC almost completely abolished chaetocin-induced apoptosis in TE-1 and KYSE150 cells (Figure 4C). Moreover, after co-treating with NAC, PARP, cleaved caspase-3, and Bcl-2 inhibited by chaetocin were reversed (Figure 4D). These results suggest that chaetocin induced the accumulation of ROS in ESCC cells, and apoptosis of ESCC cells is associated with ROS accumulation.

### The Hippo pathway is involved in chaetocin-induced ESCC cell suppresses

**Figure 5:**
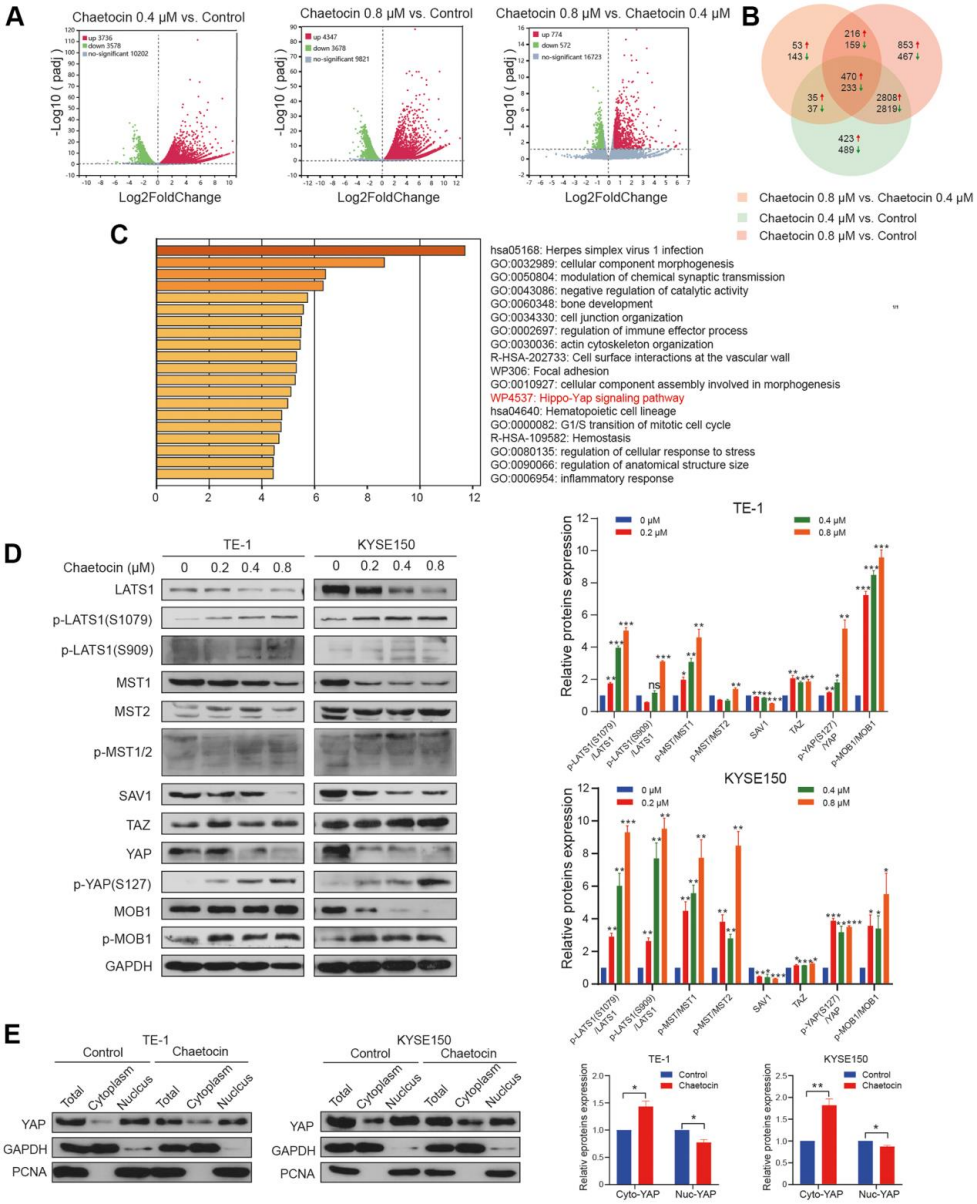
**The Hippo pathway is involved in chaetocin-induced ESCC cells apoptosis and anti-proliferation.** (**A**) RNA-seq was performed on non-treated TE-1 cells as well as TE-1 cells with 0.4 μM and 0.8 μM chaetocin treatment for 24 h. Volcano plots were used to analyze transcriptomic data, with the x-axis representing Log2FoldChange (sample/control) values and the y-axis representing the -Log10 (padj). Green, red, and gray circles respectively represent genes that were downregulated, upregulated, and not differentially regulated. (**B**) Venn diagrams demonstrating the numbers of up- and down-regulated transcripts associated with each treatment. (**C**) KEGG pathway enrichment analysis of DEGs that were specifically downregulated in the chaetocin treatment. (**D**) Representative western blot results showing changes in phosphorylation level of proteins in the Hippo pathway, including MST1/2, MOB1, LATS1, SAV1, and YAP, after chaetocin treatment. GAPDH was used as the loading control. (**E**) Cytosolic and nuclear proteins of TE-1 and KYSE150 cells treated with 0.8 μM chaetocin for 24 h were separated to detect expression levels of YAP. GAPDH and PCNA were used as the loading controls.

To fully explore the detailed molecular mechanism underlying chaetocin-mediated apoptosis and anti-proliferation effects, we conducted an RNA-seq analysis of chaetocin-treated TE-1 cells following treatments with 0.4 μM or 0.8 μM chaetocin for 24 h. After normalization and gene filtering, a total of 3736, 4347 and 774 up-regulated genes and 3578, 3678 and 572 down-regulated genes were identified in chaetocin (0.4 μM) vs. control, chaetocin (0.8 μM) vs. control, and chaetocin (0.8 μM) vs. chaetocin (0.4 μM) respectively (Figure 5A). We then assessed and categorized these differentially expressed genes (DEGs) in the different groups using Venn diagrams, which indicated that 470 up-regulated and 233 down-regulated genes were shared among the three groups (Figure 5B). Kyoto Encyclopedia of Genes and Genomes (KEGG) analysis showed that several pathways were affected by chaetocin treatment, and one of the most enriched pathways was the Hippo pathway (Figure 5C). As the Hippo pathway plays a pivotal role in esophageal squamous cell carcinoma development, we examined the Hippo-YAP pathway in relation to chaetocin-mediated cell death.

To elucidate the role of the Hippo signaling pathway in the enhanced anti-ESCC effect of chaetocin treatment, we examined the expression of central Hippo pathway molecules expression by immunoblotting using lysates of TE-1 and KYSE150 cells treated with chaetocin for 24 h. After chaetocin treatment, the phosphorylation levels of almost all core proteins in the Hippo pathway were elevated, such as MST1 (Thr183), MST2 (Thr180), MOB1 (Thr35), LAST1 (Thr1079 and Ser909), and YAP (Ser127), while total proteins were decreased (Figure 5D). Furthermore, chaetocin treatment was associated with suppressed levels of total YAP protein, which may be related to decreased nuclear translocation of the protein (Figure 5E). These results suggest that chaetocin might exhibit anticancer effects in ESCC cell via activating the Hippo pathway.

**Figure 6:**
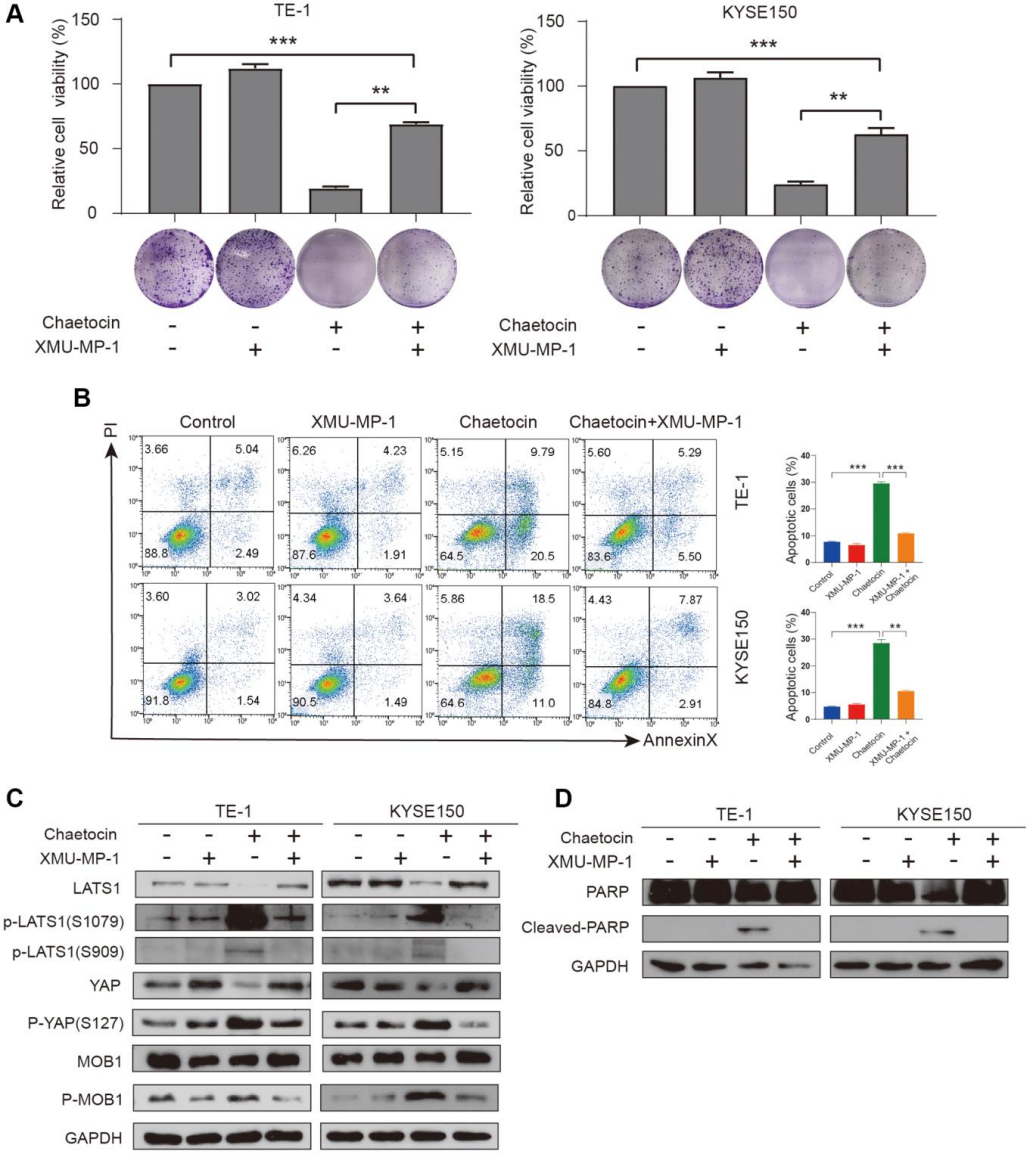
**XMU-MP-1 reverses inhibition and apoptosis of TE-1 and KYSE150 cells proliferation by chaetocin.** (**A**) TE-1 and KYSE150 cells were treated with chaetocin (0.8 μM) in the presence and absence of 1 μM XMU-MP1. The cell viability was examined by CCK-8. The colony formation was evaluated after 7 days. (**B**) Apoptosis was measured by flow cytometry. Results were shown as mean ± SD of three independent experiments. ^**^P < 0.01, ^***^P < 0.001. (**C**) Expression levels of Hippo pathway proteins were analyzed through western blot. GAPDH was used as the loading control. (**D**) Expression levels of PARP, Cleaved PARP were detected by western blot. GAPDH was used as the loading control.

To further verify whether chaetocin exhibits anticancer effects via Hippo pathway in ESCC cells, TE-1 and KYSE150 cells were treated with the Mst1/2 inhibitor XMU-MP-1 (1 μM) for 24 h before chaetocin treatment. XMU-MP-1 not only partially reversed the inhibitory effect chaetocin-induced cell proliferation, but also rescued the chaetocin-induced apoptosis in ESCC cells (Figure 6A, 6B). The phosphorylation levels of LATS1 (Ser1079 and Ser909), MOB1 (Thr35), and YAP (Ser127) were significantly reversed by XMU-MP-1, while total protein was increased (Figure 6C). Furthermore, lower levels of cleaved PARP in the XMU-MP-1 treatment group may indicate that the Mst1/2 inhibitor prevented the activation of apoptotic pathway, and rescued TE-1 and KYSE150 cells from chaetocin-induced apoptosis (Figure 6D). These data suggest that chaetocin exerts anticancer effects against ESCC via activating the Hippo pathway.

### Chaetocin suppresses the growth of ESCC cell xenografts

**Figure 7:**
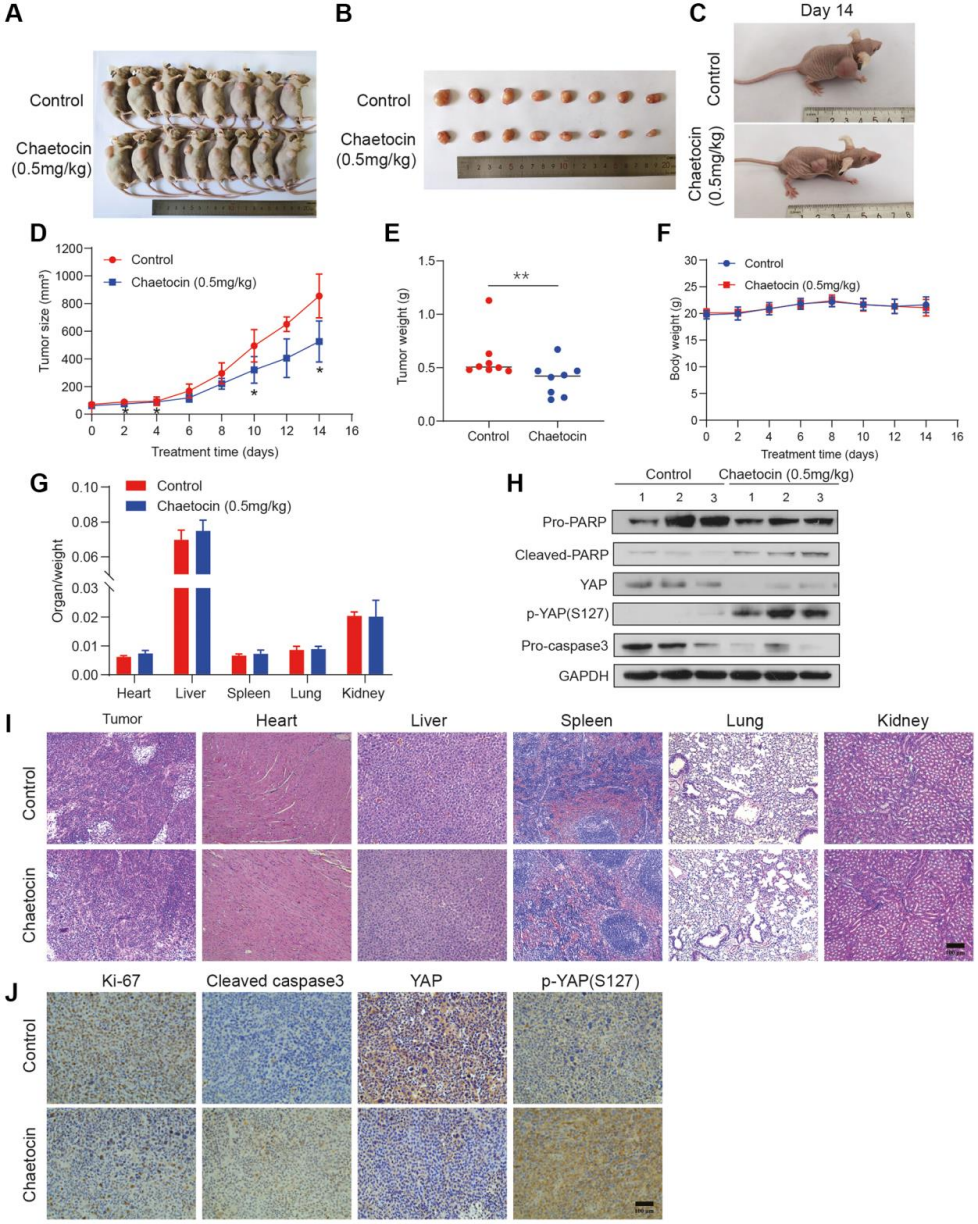
**Chaetocin suppresses the growth of ESCC cell xenografts.** (**A**) Representative photographs of a nude mouse model of xenograft tumors after control (vehicle), chaetocin (0.5 mg/kg) treatments were administered i.p. daily for 14 days. (**B**) Images of the tumor samples from each group. (**C**) The tumors of control mice and chaetocin-treated (0.5 mg/kg) mice on Day 15 are shown. (**D**) Tumor volumes were measured and calculated every other day. (**E**) Tumor mass was calculated. Error bars represent SD. ^***^P < 0.001 compared with the control using one-way analysis of variance. (**F**) Body weight during administration of chaetocin. (**G**) Relative organ weights after two weeks of treatment with chaetocin. (**H**) Western blot was performed to detect the protein expression of PARP, Cleaved PARP, YAP, p-YAP (S127), and caspase-3 in xenograft tumor. GAPDH was used as the loading control. (**I**) Representative images of H&E staining for pathological examination of different tissues. (**J**) Immunohistochemical staining for Ki67, cleaved caspase-3, YAP and p-YAP (S127) in tumor sections. Data are expressed as mean ± SD (*n* = 8). ^*^P < 0.05, ^**^P < 0.01 compared with the control group.

To further explore the potential inhibitory effects of chaetocin on ESCC growth *in vivo*, we established a subcutaneous implanted tumor model in BALB/c nude mice. Seven days after implantation of KYSE150 cells, mice were randomly divided into two groups with 8 mice in each group. Mice in the control group were administered DMSO, and mice in the experimental group received chaetocin every day, both groups were given continuous intraperitoneal injection administration for 14 d. According to previous reports, 0.5 mg/kg/d was selected as the chaetocin administration dose [27]. Remarkably, chaetocin significantly inhibited tumor growth *in vivo*, significantly decreasing tumor volume and weight compared to the control group (Figure 7A–7E). There was no significant change in body weight during the 14 d of administration (Figure 7F). The relative weight of vital organs, including the heart, liver, spleen, lungs and kidneys, did not change significantly as well (Figure 7G). Moreover, H&E staining results showed no histological differences in the heart, liver, spleen, lungs and kidneys, indicating that a range of dosages of chaetocin is not toxic but can significantly retard the growth of ESCC cell xenografts (Figure 7I). Consistent with our *in vitro* results, tumor tissues in chaetocin-treated group exhibited decreased PARP, caspase-3 and YAP protein levels, while levels of cleaved PARP and p-YAP (Ser127) were increased (Figure 7H). Immunohistochemical staining showed increased levels of both cleaved caspase-3 and p-YAP (Ser127), whereas levels of Ki-67 and YAP were decreased in chaetocin treated mice (Figure 7J). These results are consistent with those observed *in vitro*, suggesting that chaetocin inhibits the growth of ESCC and activates the Hippo pathways both *in vitro* and *in vivo*.

## DISCUSSION

ESCC is one of the most common and deadly cancers worldwide [1]. Pharmaceutical therapies such as chemotherapy, targeted therapy and immunotherapy are essential for ESCC treatment [28]. However, drug resistance is a major obstacle to achieving maximum therapeutic benefit for ESCC patients, as many patients have inherent or adaptive resistance to existing therapeutic agents [28]. Therefore, there is an urgent need to discover and develop novel agents to treat ESCC, and natural products, including marine natural products, remain a potentially important source of novel anticancer agents.

Chaetocin is a marine natural product belonging to the thiodiketopyrazine complexes first isolated in 1981 [29]. As we have reviewed, increasing evidence indicated that chaetocin exhibited potent anticancer activity against a diversity of human cancers, ranging from leukemia to solid tumors, both *in vivo* and *in vitro* [30]. In the present study, the results indicated that chaetocin not only inhibits the proliferation of ESCC cells, it also induces cell cycle arrest in the M phase. Moreover, chaetocin induces apoptosis of ESCC cells as well, characterized by the up-regulation of cleaved PARP, cleaved caspase-3, and Bcl-2 and the down-regulation of Bax. These findings clearly indicate that chaetocin treatment suppresses the malignant phenotype of ESCC.

The Hippo pathway has been reported to be associated with the progression of esophageal cancer. Frequent inactivation mutations of Hippo pathway have been confirmed and validated in ESCC [9, 10]. Interestingly, the poor-prognosis subtype of ESCC is characterized by the Hippo pathway inactivation [31]. YAP and TAZ, the core downstream effectors of the Hippo signaling pathway, are frequently overexpressed in ESCC patients and have become an independent predictor of poor prognosis [12, 32]. Reportedly, knockdown YAP or TAZ inhibits the growth of multiple cancer, including ESCC [12, 33, 34]. Bailey et al. used mouse genetic models and hPSC-derived 3D organoids to identify YAP as a key regulator of esophageal epithelial morphogenesis [35]. Therefore, these reports suggested that Hippo pathway may play a pivotal role in ESCC. Several small molecules have shown anti-esophageal cancer potential via the Hippo pathway *in vitro* and *in vivo*. Sun et al. reported that gallic acid inhibits the growth of esophageal cancer cells through Hippo signaling pathway [36]. Song et al. identified a novel YAP1 inhibitor with antitumor activity in esophageal adenocarcinoma [37]. Zhou et al. synthesized an arsenic nanocomplex that can make ESCC cancer cells sensitive to radiotherapy and chemotherapy by degrading YAP [38].

Our RNA sequencing results indicated that the Hippo-YAP pathway is one of the most enriched pathways after chaetocin treatment for 24 h, we therefore evaluated whether chaetocin treatment could activate the Hippo pathway in ESCC cells. Surprisingly, we found that the Hippo signaling proteins MST1/2, MOB1, LATS1, and YAP were significantly decreased after chaetocin treatment of ESCC cell lines, while the phosphorylation levels of these proteins were significantly increased, resulting in decreased nuclear translocation of YAP. These indicate that chaetocin activates the Hippo pathway primarily via phosphorylation of these key effectors. The selective Mst1/2 inhibitor XMU-MP-1 not only partially restores cell viability and rescues the apoptosis induced by chaetocin, it also reverses chaetocin-induced phosphorylation of LATS1 (Ser1079 and Ser909) and YAP (Ser127). Collectively, our data thus suggested that chaetocin activates the Hippo pathway.

Considerable evidence suggested that carcinogenesis was related to the moderate elevation of intracellular ROS levels, while the excessive accumulation of ROS in cancer cells trigged cell death. Chaetocin reportedly induces ROS accumulation, thereby significantly inhibiting the growth of cancer cells, such as gastric cancer and glioma [26, 27]. Recent studies have highlighted the relationship between oxidative stress and Hippo pathway, and shown that ROS could activate the Hippo pathway [15]. Therefore, some small molecule targeting ROS/Hippo is being investigated. Nakatani et al. reported that alantolactone inhibits YAP1/TAZ by promoting the accumulation of reactive oxygen species, thus inhibiting tumor growth [16]. Zhang et al. found that the production of intracellular ROS was significantly correlated with the antitumor activity of nitidine chloride and the down-regulation of YAP [39]. In the present study, we found that chaetocin significantly elevated intracellular ROS levels in ESCC cells as well, and the ROS scavenger NAC almost completely eliminated chaetocin induced apoptosis in ESCC cells. Moreover, chaetocin promotes intracellular ROS accumulation, which may be related to the activation of Hippo pathway.

The results of *in vivo* experiments indicated that tumor volume and tumor weight were significantly reduced after chaetocin treatment, while body weight did not change much. Moreover, no histological differences were noted in the heart, liver, spleen, lungs, and kidneys, suggesting no significant toxicity at a given dose of chaetocin in xenografts. Notably, we observed marked increases in the levels of cleaved caspase-3 and phosphorylated YAP in the chaetocin-treated group, and a dramatic decrease in the level of total YAP, which is consistent with the results of *in vitro* experiments.

**Figure 8:**
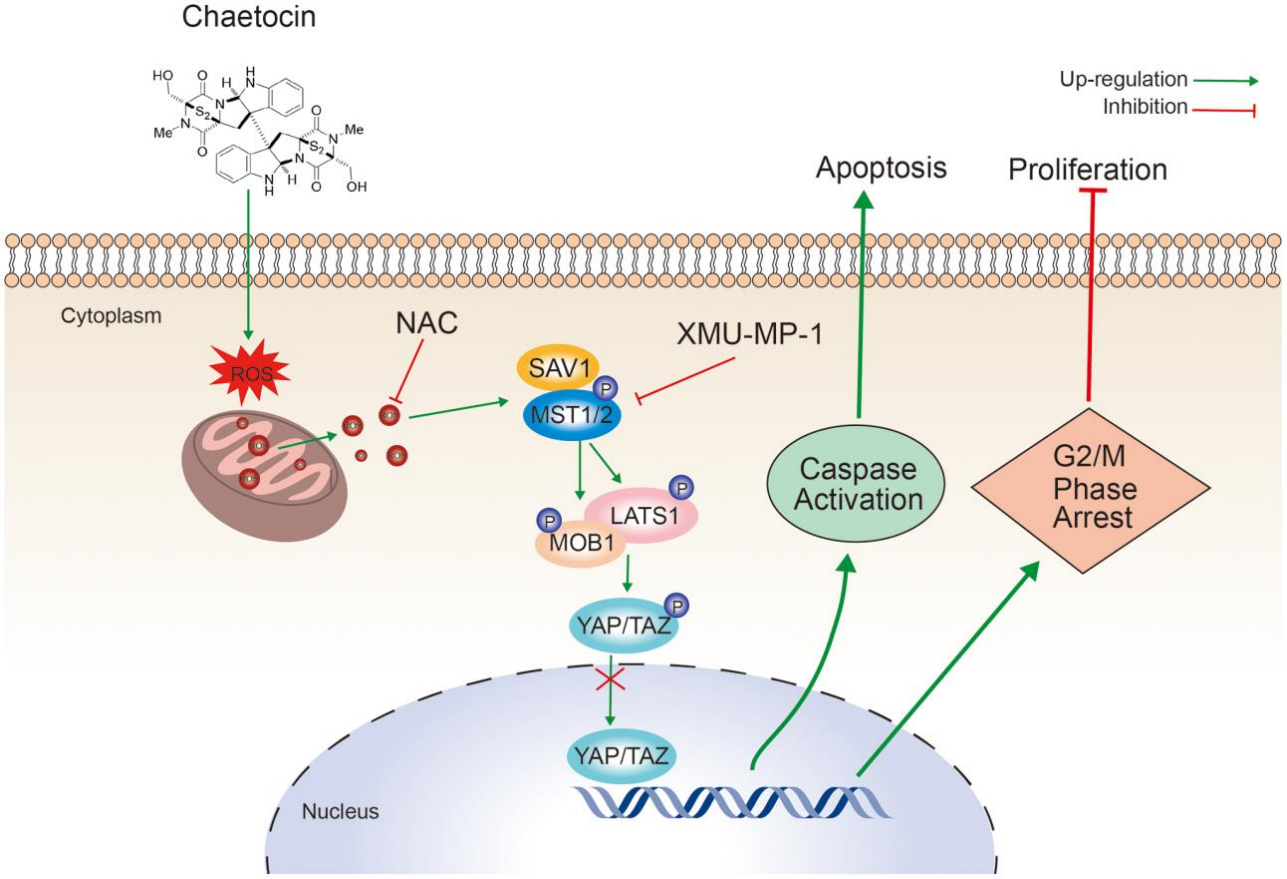
Schematic representation of the mechanism of the action of chaetocin in ESCC cells.

In summary, our data demonstrate that chaetocin could significantly inhibit ESCC cell proliferation, induce apoptosis, and induce cycle arrest in the M phase. Moreover, chaetocin could promote ROS accumulation and activate Hippo signaling pathway to exert anti-ESCC activity (Figure 8). These results provide an important basis for further research of chaetocin as a potential therapeutic or preventive candidate agent for ESCC treatment.

## Supplementary Materials

Supplementary Figure 1

Supplementary Tables
