# Supplementary material for: Chaetocin exhibits anticancer effects in esophageal squamous cell carcinoma via activation of Hippo pathway
Source: Aging (Albany NY). 2023 Jun 14;15(12):5426–44. doi: 10.18632/aging.204801 (PMC10333076; doi:10.18632/aging.204801)
Supplement: Supplementary Figure 1 [file aging-15-204801-s001.pdf]

SUPPLEMENTARY FIGURE

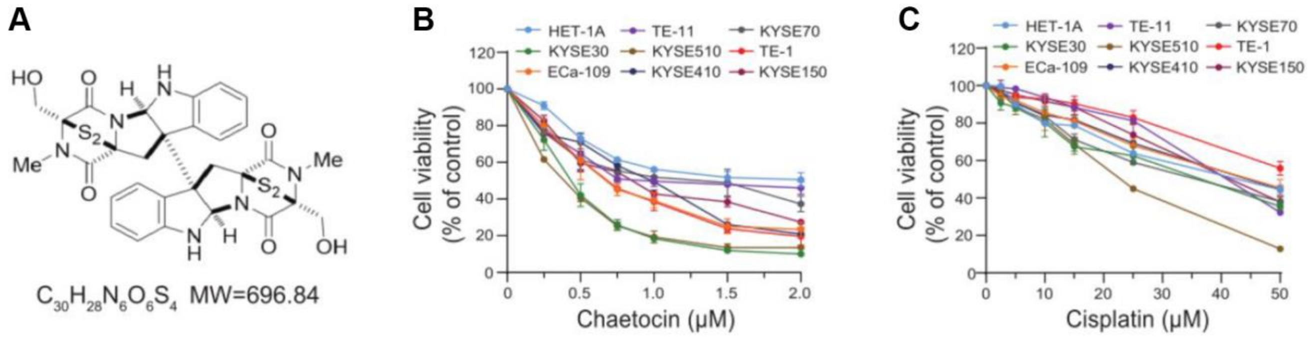

Supplementary Figure 1. Chaetocin suppresses the growth of ESCC cells. (**A**) Chemical structure of chaetocin. (**B**) Followed by treatment with chaetocin at the indicated concentrations for 24 h, CCK8 assay was used to measure the cell viability of ESCC cells. (**C**) Followed by treatment with cisplatin at the indicated concentrations for 24 h, CCK8 assay was used to measure the cell viability of ESCC cells.
