# Supplementary material for: Chaetocin exhibits anticancer effects in esophageal squamous cell carcinoma via activation of Hippo pathway
Source: Aging (Albany NY). 2023 Jun 14;15(12):5426–44. doi: 10.18632/aging.204801 (PMC10333076; doi:10.18632/aging.204801)
Supplement: Supplementary Tables [file aging-15-204801-s002.pdf]

SUPPLEMENTARY TABLES

Supplementary Table 1. Effects on viability of chaetocin to ESCC cells and HET-1A.

Cell line	IC50 (μM)
Het-1A	1.62
KYSE30	0.75
KYSE410	1.06
KYSE510	0.36
KYSE70	1.68
ECa-109	1.36
TE-1	0.67
TE-11	1.21
KYSE150	0.85

Supplementary Table 2. Effects on viability of cisplatin to ESCC cells and HET-1A.

Cell line	IC50 (μM)
Het-1A	39.16
KYSE30	30.36
KYSE410	42.13
KYSE510	19.71
KYSE70	33.49
ECa-109	42.62
TE-1	60.19
TE-11	39.04
KYSE150	39.95
